# Hepatic ketogenesis regulates lipid homeostasis via ACSL1-mediated fatty acid partitioning

**DOI:** 10.21203/rs.3.rs-3147009/v1

**Published:** 2023-07-18

**Authors:** Sadeesh Ramakrishnan, Raja Gopal Reddy Mooli, Yerin Han, Ericka Fiorenza, Suchita Kumar, Fiona Bello, Anoop Nallanagulagari, Shreya Karra, Lihong Teng, Michael Jurczak

**Affiliations:** University of Pittsburgh; University of Pittsburgh; University of Pittsburgh; University of Pittsburgh; University of Pittsburgh; University of Pittsburgh; University of Pittsburgh; University of Pittsburgh; University of Pittsburgh; University of Pittsburgh

## Abstract

Liver-derived ketone bodies play a crucial role in fasting energy homeostasis by fueling the brain and peripheral tissues. Ketogenesis also acts as a conduit to remove excess acetyl-CoA generated from fatty acid oxidation and protects against diet-induced hepatic steatosis. Surprisingly, no study has examined the role of ketogenesis in fasting-associated hepatocellular lipid metabolism. Ketogenesis is driven by the rate-limiting mitochondrial enzyme 3-hydroxymethylglutaryl CoA synthase (HMGCS2) abundantly expressed in the liver. Here, we show that ketogenic insufficiency via disruption of hepatic HMGCS2 exacerbates liver steatosis in fasted chow and high-fat-fed mice. We found that the hepatic steatosis is driven by increased fatty acid partitioning to the endoplasmic reticulum (ER) for re-esterification via acyl-CoA synthetase long-chain family member 1 (ACSL1). Mechanistically, acetyl-CoA accumulation from impaired hepatic ketogenesis is responsible for the elevated translocation of ACSL1 to the ER. Moreover, we show increased ER-localized ACSL1 and re-esterification of lipids in human NASH displaying impaired hepatic ketogenesis. Finally, we show that L-carnitine, which buffers excess acetyl-CoA, decreases the ER-associated ACSL1 and alleviates hepatic steatosis. Thus, ketogenesis via controlling hepatocellular acetyl-CoA homeostasis regulates lipid partitioning and protects against hepatic steatosis.

## Introduction

Non-alcoholic fatty liver disease (NAFLD) is one of the most heterogeneous forms of liver disease, with a prevalence of ~ 24% in the United States and rapidly rising worldwide. Hepatic steatosis or fatty liver is the first hit in NAFLD pathogenesis, wherein lipotoxicity from the accumulated lipids induces oxidative stress, insulin resistance, inflammation, and fibrosis, leading to the development of non-alcoholic steatohepatitis (NASH). About 20% of NAFLD patients progress to end-stage liver diseases, such as cirrhosis and hepatocellular carcinoma, increasing overall mortality. Despite the increasing incidence and mortality, no FDA-approved drugs are available due to the incomplete understanding of NAFLD pathogenesis^1,2 3,4^. Moreover, the links between lipid metabolism and NALFD pathogenesis in response to energy demands are not well defined. Hepatic lipid accumulation is determined by a mismatch in free fatty acid uptake, *de novo* lipogenesis, fatty acid oxidation, and very low-density lipoprotein (VLDL) secretion^5,6^. In this process, lipid partitioning plays an essential role in adapting to systemic energy needs^7^. For instance, free fatty acids entering the hepatocytes are partitioned toward mitochondrial fatty acid oxidation (FAO) at fasting. In contrast, fatty acids are targeted to the endoplasmic reticulum (ER) for esterification and storage as lipid droplets under nutrient-rich conditions^7^. An imbalance in lipid partitioning induces lipid accumulation leading to hepatic steatosis and injury, the prerequisite step for the development and progression of NAFLD and its progressive form NASH^7,8^. Despite the significance of fatty acid partitioning in hepatic lipid homeostasis, the mechanisms that govern them are under-studied.

Fasting is the appropriate model to dissect the mechanism of lipid partitioning as the latter dictates FAO in a controlled manner. Fasting is characterized by adipose tissue lipolysis, increased delivery of fatty acids to the liver, and enhanced FAO ^9,10^. Hepatic FAO generates acetyl-CoA that is preferably converted into ketone bodies via the mitochondrial 3-hydroxymethulglutaryl-CoA synthase 2 (HMGCS2), the rate-limiting ketogenic enzyme highly expressed in the hepatocytes^11^. Ketone bodies secreted from the liver fuel the peripheral organs such as the brain and muscle^12,13^. Recent studies show that neonates with ketogenic insufficiency develop fatty liver, which could be rescued by early weaning^14,15^. Similarly, feeding a high-fat diet to mice with ketogenic insufficiency induces hepatic steatosis and liver injury^13,15,16^. These data indicate that ketogenesis is a conduit to remove excess dietary lipids, thereby protecting against fatty liver. During fasting, hepatocytes are bombarded with a huge influx of adipose tissue-derived fatty acids^17^. Although ketogenesis is known to be induced during fasting^17^, its significance in regulating hepatocellular lipid homeostasis is not well understood.

We use conditional liver-specific HMGCS2 knockout mice to report that ketogenesis insufficiency exacerbates fatty liver in chow and high-fat feeding mice at fasted state. Impaired ketogenesis did not affect fatty acid uptake, *de novo* lipogenesis, and β-oxidation but increased fatty acid storage as lipid droplets. Incoming fatty acids are esterified into acyl-CoA by the enzyme Acyl-CoA synthetase long-chain family member 1 (ACSL1) localized on mitochondria and ER^18,19^. Mitochondrial ACSL1 promotes β-oxidation, whereas ER-associated ACSL1 favors lipid storage via re-esterification^20^. The mechanisms regulating the ER localization of ACSL1 and its implication in hepatic lipid metabolism are not well understood. We show that acetyl-CoA accumulation from ketogenic insufficiency induces ER translocation of ACSL1, resulting in fatty acid re-esterification and steatosis. We report that a similar mechanism exists in human NASH livers, wherein a reduction in HMGCS2 is associated with increased ER-associated ACSL1. Excessive acetyl-CoA is buffered by L-carnitine, and this buffering capacity is diminished in NASH due to acquired carnitine deficiency^21,22^. We show that hepatic L-carnitine levels were significantly lower in ketogenic insufficient mice, and restoring L-carnitine attenuated fasting-induced acetyl-CoA accumulation, ER-ACSL1, and hepatic steatosis. We also demonstrate that the lipid-lowering effect of L-carnitine is associated with a reduction in the ER-associated ACSL1 in primary hepatocytes derived from NASH patients. Overall, our study defines the crucial role of hepatic ketogenesis in lipid homeostasis by regulating the partitioning of fatty acids via the acetyl-CoA-ER-ACSL1 axis.

## Materials and Methods

### Animal studies

We generated a *Hmgcs2*^F/F^ founder line on a C57BL6 background by CRISPR/Cas-9 mediated genome engineering, wherein exon 2 was floxed. Mice with conditional knockout of HMGCS2 (*Hmgcs2*^ΔLiv^ mice) were generated by crossing *Hmgcs2*^F/F^ mice with mice carrying tamoxifen-inducible Cre recombinase under the control of Albumin promoter (Alb-Cre/ERT2). Littermates that do not express Cre recombinase were used as the control. Tamoxifen (Cayman Chemical, Ann Arbor, MI) was dissolved at 10mg/ml in corn oil (Sigma-Aldrich, St. Louis, MO) and administered intraperitoneally at a dose of 100mg/kg body weight for two consecutive days. All the experiments were carried out at least two weeks after tamoxifen injection. All the animals were maintained on the chow diet provided by the DLAR. For high fat studies, mice were fed with an HFD (60% kcal from fat, D12492; Research Diets) diet for 4 weeks. Mice euthanized at the fed state or after overnight fast (6.00 PM-10.00 AM). For the L-carnitine experiment, 6–8-week-old *Hmgcs2*^ΔLiv^ mice were provided with drinking water containing L-Carnitine (@10mg/ml TCI, Portland, OR; C0049) 9 hours before fasting. All the mice were maintained at a 12-hour light/dark cycle with free access to food and water. All experiments were performed using age- and sex-matched littermates, unless indicated in the figure legends. All animal experiments were approved by the Animal Care and Use Committee at the University of Pittsburgh.

### Mouse primary hepatocyte isolation

Mouse primary hepatocytes were isolated from 6–8-week-old *Hmgcs2*^ΔLiv^ male mice. Briefly, mice were anesthetized using isoflurane, and the inferior vena cava was cannulated and infused with 10 ml of perfusion buffer (HBSS with no Ca^2+^, no Mg^2+^, no phenol red, supplemented with 0.5 mM of EDTA and 25 mM of HEPES, pH 7.4) with a flow rate of 3ml/min. Then, 10 ml of digestion buffer (HBSS with no Ca^2+^, no Mg^2+^, no phenol red, supplemented with 25 mM of HEPES, pH 7.4) with collagenase type 1 (15mg/50ml) was infused using a peristaltic pump. The liver was excised, minced, and filtered using a 70 μm cell strainer (Falcon) and centrifuged at 50 × g for 2 min to pellet hepatocytes. Dead cells were removed by centrifugation at 50 × g for 10 min in 90% Percoll solution (Sigma-Aldrich) in pre-chilled 10X PBS. Cells were resuspended in DMEM (Gibco) supplemented with 10% fetal bovine serum (FBS) and 1% penicillin/streptomycin. The cells were counted and plated onto the 12-well or 10 cm plates at a cell density of 0.2 ×10^6^cells/ml. After 4 hours of incubation, the cells were refreshed with Williams E media supplemented with 10% fetal bovine serum (FBS) and 1% penicillin/streptomycin. For Triascin C treatment, hepatocytes were incubated with 200μM bovine serum albumin-conjugated palmitate (BSA-PA; Cayman Chemical) and treated with 5μM Triascin C (Enzo Life Sciences, Farmingdale, NY) or DMSO vehicle for 16 hours. For the acetate treatment, mouse primary hepatocytes were incubated with 200μM BSA-PA or BSA and treated with 20 mM sodium acetate (Sigma-Aldrich) for 16 hours.

### Human NASH subjects and primary hepatocyte isolation

A total of 20 human subjects (10 NASH and 10 Normal) were scheduled for a liver biopsy to diagnose NASH, and were recruited by participating physicians at the University of Pittsburgh Medical Center (Pittsburgh, PA, USA). All the patients fulfilled the inclusion criteria, such as no positive for viral hepatitis, Wilson disease, or any other possible cause of liver dysfunction. The patients were excluded if the alcohol consumption exceeded 20 g/week. Primary hepatocytes were isolated from explanted human liver segments obtained from patients receiving orthotopic liver transplantation for decompensated liver cirrhosis due to NASH. The specimens were obtained by the Human Synthetic Liver Biology Core at the Pittsburgh Liver Research Center (PLRC) using a protocol approved by the Human Research Review Committee and the Institutional Review Board (IRB STUDY20090069) at the University of Pittsburgh. Liver tissue specimens were protected from ischemic injury by flushing with ice-cold University of Wisconsin (UW) solution immediately after resection in the operating room, keeping the specimens on ice, and transporting the specimens immediately to the laboratory. Hepatocytes were isolated by a modified three-step perfusion technique. Briefly, the livers were flushed under a sterile biosafety hood through the hepatic vessels (re-circulation technique) with pre-warmed calcium-free HBSS (Sigma, H6648-1L) supplemented with 0.5 mM EGTA (Thermo Fisher, 50-255-956) and then with collagenase/protease solution (VitaCyte, 007–1010) until the tissue was fully digested. The digestion time for each preparation was in the range of 45–60 min. The digested liver was removed and immediately cooled with ice-cold Leibovitz’s L-15 Medium (Invitrogen, 11415114) supplemented with 10% FBS (Sigma, F4135). The final cell suspension was centrifuged twice at 65xg for 7 min at 4°C and the medium was aspirated. The yield and viability of freshly isolated hepatocytes were estimated by trypan blue staining.

### Neutral lipid staining using Oil-red-O and BODIPY

Cells were washed with 1X PBS and fixed with 10% PBS-buffered formalin at room temperature for 30 min, washed with ddH_2_O, and incubated with 60% isopropyl alcohol for 5 min. The cells were incubated in 0.5% Oil red O (Sigma-Aldrich) staining reagent for 30–45 min. Then, the cells were washed with ddH_2_O thrice and counterstained with hematoxylin for 15 sec, and washed several times with ddH_2_O. The images were captured using an EVOS microscope (Olympus, Tokyo, Japan). To quantify the intracellular lipids, the stain was extracted in 250 μl isopropanol incubated at room temperature for 10–15 min, and absorbance was measured at 492 nm. For BODIPY staining, cells were fixed with 10% PBS-buffered formalin at room temperature for 20 min, washed with 1X PBS, and stained with 250 μl of BODIPY (1mg/ml stock diluted 1:1000; Cayman Chemical) for 15–20 min. The cells were washed with PBS, and images were captured using an EVOS microscope.

### Blood and serum analysis

The tail was snipped, and blood glucose levels were measured using a glucometer (Bayer, Parsippany, NJ). Serum β-hydroxybutyrate levels (Cayman Chemical, Ann Arbor, MI) were measured using a calorimetry kit following manufacturers’ instructions. Serum triglycerides and cholesterol levels were measured using colorimetric Infinity Triglyceride and Cholesterol Reagent kits (Thermofisher Scientific, Middletown, VA). Serum non-esterified fatty acid was quantified using a colorimetric assay (Fujifilm Wako Diagnostics, Lexington, MA).

### Liver triglyceride assay

Hepatic triglyceride levels were measured as described previously ^23^. Briefly, human and mouse frozen liver tissues (~50 mg) were homogenized in 3 ml of chloroform: methanol (2:1) mixture and vortexed. The homogenate was incubated for 60 min at room temperature while rotating on a shaker. Then, the homogenate was acidified with 1mol/L H_2_SO_4_, and lipid fractions in the lower organic phase were collected after centrifugation at 1300 rpm for 10 min and transferred to clean glass vials. Triglyceride levels were determined using the triglyceride reagent (Thermo Fisher Scientific) and normalized to liver weight.

### Thin layer chromatography (TLC)

Equal amount of lipids extracted from human and mouse livers were dried completely under a N-EVAP nitrogen evaporator (Organomation, Berlin MA) and resuspended with 100 μl chloroform: methanol (2:1). Before running the TLC plate, the running chamber was equilibrated with 200ml of the solvent system containing petroleum ether: ethyl ether: acetic acid mixture (25:5:1), and the TLC plate was baked at 75°C for 30 min. Aliquots of 25 μl resuspended lipid extracts and lipid standard (Nu-Chek-Prep, Elysian, MN), were loaded onto the TLC plates (EMD Millipore) and lipids were separated by the solvent system.. The plate was removed from the chamber when the solvent reached about 1 inch to the top edge of the plate, and was dried for 5–10 min (until all solvents evaporated). The plate was then transferred to an equilibrated iodine tank for about 30–45 min for staining, and images were captured with CanoScan LidE 220 imager (Canon). ImageJ software (NIH, Bethesda) was used to quantify the lipid band density.

### Liver histology

Liver tissue was excised and immediately fixed in 10% PBS-buffered formalin (Thermofisher Scientific). H&E staining was performed in 6-micron paraffin-embedded sections, and the images were captured using an EVOS microscope.

### RNA isolation, cDNA synthesis, and qPCR analysis

Total RNA was extracted using TRIzol reagent (Life Technologies) as per manual instructions. RNA was quantified using a Nanodrop, and 1 μg of RNA was reverse-transcribed using Moloney murine leukemia virus (Mu-MLV) reverse transcriptase (Promega, Madison, WI). mRNA levels were analyzed using SYBR Green PCR Master Mix (ApexBio, Houston, TX) with QuantStudio 3 Station qPCR machine (Applied Biosystems, Foster City, CA). The relative expression of target genes was calculated using a comparative delta threshold cycles (ΔCT) method after normalizing to β-actin. The primer sequences are provided in Table S1.

### Western blotting

Cells or human and mouse frozen tissues (~10mg) were homogenized using radioimmunoprecipitation assay lysis buffer (0.5% NP-40, 0.1% sodium deoxycholate, 150 mmol/L NaCl, 50 mmol/L Tris-Cl, pH 7.5) containing 1 mmol/L phenylmethylsulphonyl fluoride, protease inhibitor cocktail (Sigma-Aldrich) and 2 mmol/L sodium orthovanadate^24^. The homogenate was centrifuged at 13,000 × g for 10 min at 4°C to collect the supernatant. The protein concentration was quantified using a protein assay kit (Bio-Rad, Hercules, CA), and the sample was resolved on a SDS-PAGE. The membranes were blocked with 3% skim milk and incubated with primary antibodies, overnight at 4°C. Secondary antibodies conjugated with DyLight (Cell Signaling Technology) were added to the membranes (antibody details are in Table S2) and visualized using the Odyssey CLx Imaging System (LI-COR, Lincoln, NE).

### Subcellular fractionation for western blot

Mitochondrial and microsomal fractions were isolated as described previously^20^. In brief, cell pellet and liver tissues from mice and humans were minced in mitochondrial isolation buffer (MSHE, 70 mM sucrose, 210 mM mannitol, 5 mM HEPES, 1mM EGTA, pH 7.2 with 2% fatty acid-free BSA) with protease and phosphatase inhibitors. The minced tissue was homogenized by stroking 25–30 times using a Teflon glass homogenizer on ice. The homogenate was transferred to the Eppendorf tube and centrifuged at 800 × g for 10 min, and the supernatant was centrifuged at 8000 × g for 10 min to pellet the mitochondria. The mitochondrial pellet was washed by resuspending in MSHE buffer and centrifuged at 8000 × g for 10 min. The microsomal fraction was collected by transferring the supernatant of the mitochondrial pellet to a new tube containing 8 mM of CaCl_2_ and incubated on a rotating platform in the cold room for 10 min. The microsomal pellet was collected by centrifuging at 30000 × g for 30 min. The mitochondrial and microsomal pellets were resuspended in RIPA buffer containing protease and phosphatase inhibitor cocktail.

### Crude mitochondria isolation for respirometry

A half lobe of the freshly sampled liver was transferred to ice-cold PBS, cut into small pieces, and washed three times with PBS. 200 mg of the liver was weighed and transferred to 2mL of SMET buffer (10 mM Tris HCl (pH 7.5), 220 mM Mannitol, 70 mM Sucrose, 1 mM EDTA, 0.25% BSA) and homogenized using a glass dounce and rotating teflon pestle, applying eight strokes at max speed. Homogenates were centrifuged at 800× g for 10 min at 4°C to discard cell debris and nuclei. The supernatant was collected, avoiding the top fat layer, and centrifuged again to eliminate remaining cell debris and nuclei. A fraction of the supernatant was saved for protein assays and citrate synthase activity measurement. The remaining supernatant was then centrifuged at 8000 × g for 10min at 4°C to pellet crude mitochondria. Pellets were resuspended in 400μL SMET buffer, and 80μL was used for respirometry assays. Remaining pellet suspension was centrifuged 8000 × g for 10min at 4°C and saved for protein quantification. Mitochondrial respiratory capacity was assessed using an Oroboros O_2_K High-Resolution Respirometer and MiR05 buffer at 37°C under constant mixing in a sealed, 2-ml chamber. Respirometry assays were performed to determine respiration devoted to ATP synthesis, maximum or uncoupled electron transport chain capacity and non-mitochondrial respiration. Membrane integrity was assessed by the addition of cytochrome c after addition of ADP. Substrate and inhibitor concentrations were as follows: palmitoylcarnitine (40 μM), malate (2 mM); adenosine diphosphate (ADP, 4 mM); cytochrome c (10μM), FCCP (1μL titration) and antimycin A (2.5 μM). The protein concentration of the crude mitochondria was determined by BCA protein assay and used to express respiratory capacities as oxygen consumption per protein mass (pmol s^− 1^ mg^− 1^). Respiration in the presence of antimycin A was subtracted from all respiratory rates to account for non-mitochondrial respiration.

### Acetyl-CoA measurement

The acetyl-CoA levels were measured using a fluorometric PicoProbe Acetyl CoA assay kit (Abcam, Waltham, MA) following manufacturers’ instructions. Human and mouse liver tissue (~25mg) was homogenized in 1N perchloric acid (PCA) and centrifuged at 10,000 × g for 10 min at 4°C. The samples were neutralized with 3M KHCO_3_ to pH ~ 6–8 while keeping on ice. The supernatant was collected by centrifuging at 13,000 × g for 15 min at 4°C. The assay was carried out per the manual instructions, and the final concentration was adjusted based on the sample dilution factor and tissue weight.

### L-carnitine measurement

L-carnitine levels were measured by colorimetric L-carnitine assay kit (Abcam, Waltham, MA) according to manufactures instructions. Human and mouse frozen liver tissues (~25mg) were homogenized with 250 μl assay buffer and the supernatant was collected by centrifuging at 13,000 × g for 10 min. The samples were deproteinized by adding ice-cold perchloric acid (PCA) to a final concertation of 1 M and mixed well. After incubating on ice for 5 min, the samples were centrifuged at 13,000 × g for 2 min at 4°C. The supernatant was neutralized to pH 6.5–8 using ice-cold 2 M KOH. Finally, the supernatant was collected for the assay by centrifuging at 13,000 × g for 15 min at 4°C. The assay was carried out as per the manual instructions and the final concentrations of the L-carnitine were adjusted for the sample dilution factors and tissue weight.

### Body composition analysis

Body composition was assessed in conscious mice using EchoMRI (EchoMRI LLC, TX, USA). Percent fat and lean mass were calculated for individual mice by dividing the weight of the tissue determined by EchoMRI by the body weight.

### Quantification and Statistical data analysis

The data is presented as mean ± SEM. Statistical analyses were performed using Prism software version 9.0.0 (GraphPad Software). Comparisons between the two groups were made by conducting Student *t*-tests. One-way ANOVA (Tukey multiple-comparisons test) was used to compare more than two groups. Differences were considered statistically significant if p < 0.05 (*); p < 0.01 (**) or p < 0.001 (***) or p < 0.0001(****). The band intensities for western blot and TLC were quantified using Image J 1.52q. The statistical methods of each experiment are indicated in the figure legends. *In vitro* experiments were replicated at least three independent times, except for human NASH hepatocytes.

## Results

### Hepatic ketogenesis protects against fasting-induced hepatic steatosis.

**Figure 1:**
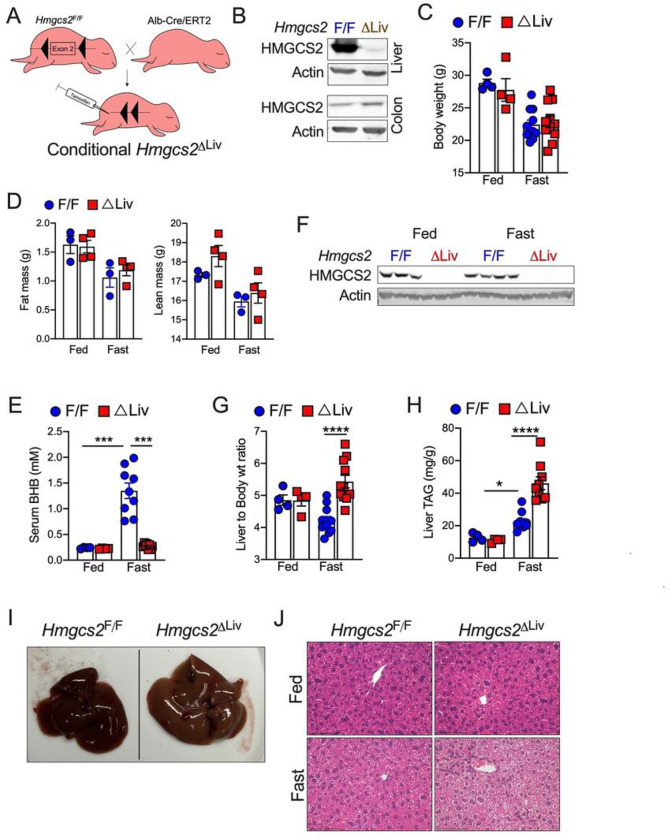
Hepatic ketogenesis protects against fasting-induced hepatic steatosis. (A) Schematic representation showing the generation of conditional liver-specific HMGCS2 knockout mice. (B) WB analysis showing HMGCS2 protein expression in the liver and colon of *Hmgcs2*^ΔLiv^ mice. (C) Body weight in fed or 16 h fasted *Hmgcs2*^ΔLiv^ mice. (D) Fat mass and lean mass determined by NMR. (E) Serum b-hydroxybutyrate (BHB) levels in fed and fasted *Hmgcs2*^ΔLiv^ mice. (F) WB analysis showing HMGCS2 protein expression in the liver of fed and fasted *Hmgcs2*^ΔLiv^ mice. (G) Liver-to-body weight ratio, (H) Hepatic total triglyceride (TAG), (I) Gross image, and (J) H&E analysis of the livers (Magnification 20X) from fed or 16 h fasted *Hmgcs2*^F/F^ and *Hmgcs2*^ΔLiv^ mice. All the data is presented as mean ± SEM. p < 0.05 (*) or p < 0.001 (***) or p < 0.0001 (****) analyzed by One-way ANOVA (Tukey multiple-comparisons test).

Genetic studies dissecting the role of ketone bodies were performed in mice with constitutive knockout of HMGCS2 (using albumin promoter-driven Cre recombinase) in the liver. However, these mice develop severe hepatic steatosis and mitochondrial dysfunction at the neonatal age^14^. To assess the role of hepatic ketogenesis in metabolic homeostasis without any confounding developmental defects, we generated a mouse model with conditional knockdown of HMGCS2 in the liver (*Hmgcs2*^ΔLiv^) by crossing our *Hmgcs2*-floxed mice (*Hmgcs2*^F/F^) with mice expressing tamoxifen-inducible cre under the control of albumin promoter (Fig. 1A). Administration of tamoxifen decreased HMGCS2 protein expression in the liver but not in other organs such as the colon, kidney, and heart (Fig. 1B and S1A), which are also minimally involved in ketone body production^12^. Note, littermate *Hmgcs2*^F/F^ mice administered with tamoxifen were used as control throughout the studies. Ketone bodies play a crucial role in fasting energetics^12^; therefore, we assessed *Hmgcs2*^F/F^ and *Hmgcs2*^ΔLiv^ mice under fed and 16-hours fasted conditions. Fasting decreased body weight, fat mass, and lean mass with no difference between the genotypes (Fig. 1C and D). As expected, fasting significantly increased the serum ketone body, β-hydroxybutyrate (BHB) levels in *Hmgcs2*^F/F^ mice (Fig. 1E), though no difference in hepatic HMGCS2 protein expression was noticed (Fig. 1F). Disruption of HMGCS2 in the liver significantly decreased ketone body levels in *Hmgcs2*^ΔLiv^ mice (Fig. 1E), consistent with a recent report that ketone bodies are majorly derived from the liver^25^. Ketone body metabolism in peripheral tissues, particularly muscle, increases circulating glucose levels as ketone body oxidation competes with glucose utilization^26^. However, inhibiting hepatic ketogenesis did not affect the fasting glucose levels under nutrient-deprived conditions (Fig S1B).

Previous studies showed that constitutive deletion of hepatic HMGCS2 results in fatty liver in postnatal mice pups (as early as 4 days after birth)^14,15^. However, anti-sense oligomers (ASO)-mediated knockdown of Hmgcs2 in adult mice did not affect the hepatic lipid content under fed conditions^16^. Similarly, temporal disruption of Hmgcs2 in adult mice did not affect liver-to-body weight ratio or hepatic triglyceride content in *Hmgcs2*^ΔLiv^ mice under fed conditions (Fig. 1G and H). However, fasting increased the liver-to-body weight ratio with paler liver in *Hmgcs2*^ΔLiv^ mice (Fig. 1G and I). In line with that, liver triglyceride (TAG) levels were significantly elevated in fasted *Hmgcs2*^ΔLiv^ mice (Fig. 1H). Further, H&E analysis revealed microsteatosis in the fasted *Hmgcs2*^ΔLiv^ livers (Fig. 1J). Serum triglycerides were similar between the fed and fasted mice (Fig S1C). We found a trend towards an increase in serum cholesterol levels in *Hmgcs2*^ΔLiv^ mice; however, fasting did not affect cholesterol levels in both genotypes (Fig S1D). Together, our data suggest that impaired ketogenesis induces hepatic steatosis under nutrient-deprived conditions.

### Ketogenic insufficiency induces hepatic steatosis via ACSL1.

**Figure 2:**
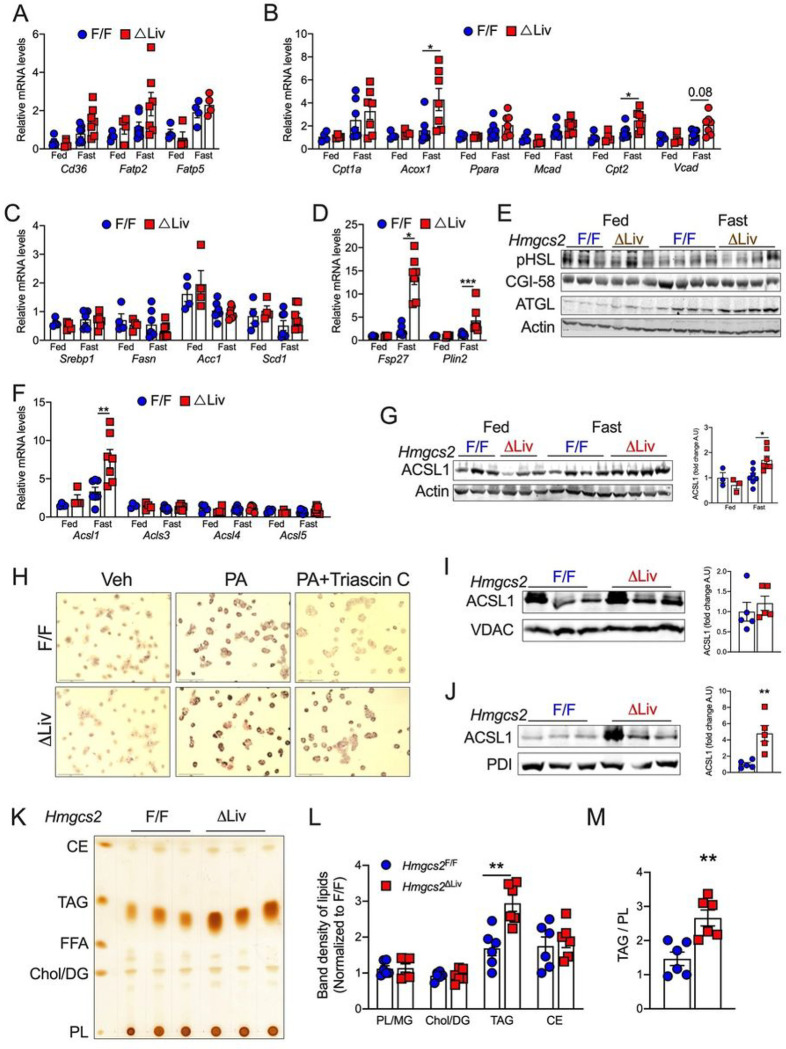
Ketogenic insufficiency increases ER-associated ACSL1 leading to fatty acid partitioning toward esterification. (A-D) Relative mRNA levels of genes involved in (A) fatty acid uptake, (B) fatty acid oxidation, (C) *de novo* lipogenesis, and (D) Lipid storage/droplet-associated proteins in the livers of fed or 16 h fasted *Hmgcs2*^ΔLiv^ mice. (E) WB analysis of lipolysis-associated proteins in the liver lysates from *Hmgcs2*^ΔLiv^ mice. (F) Relative mRNA levels of ACSL1 isoforms in the livers of *Hmgcs2*^ΔLiv^ mice. (G) WB analysis and quantification of ACSL1 in the liver of *Hmgcs2*^ΔLiv^ mice. (H) Oil-red-O staining of primary hepatocytes from *Hmgcs2*^ΔLiv^ mice and *Hmgcs2*^F/F^ mice loaded with 200 mM BSA-PA in the presence or absence of 5mM Triascin C treatment for 16 h (20X magnification). (I and J) WB analysis of ACSL1 in the hepatic mitochondrial and microsomal fractions from 16 h fasted *Hmgcs2*^F/F^ mice. (K-M) Thin layer chromatography (TLC) image showing lipid fractions including Phospholipids (PL), Cholesterol/Diacylglycerol (Chol/DG), free fatty acids (FFA), Triacylglycerol (TAG) and ceramides (CE). (L) Quantified band densities for TAG lipid fractions and (M) Quantified ratio of TAG/PL in the liver of fasted *Hmgcs2*^F/F^ mice. All the data is presented as mean ± SEM. p < 0.05 (*) or p < 0.01 (**) or p < 0.001 (***) analyzed by One-way ANOVA (Tukey multiple-comparisons test) and Two-tailed Student *t*-test.

During fasting, non-esterified fatty acids (NEFA) released from the adipose tissue via lipolysis enter the liver to undergo FAO^27^. An increase in adipose tissue lipolysis could induce hepatic steatosis^27^. Adipose tissue levels of lipolysis-related proteins, such as pHSL and ATGL (Fig S2A), and the circulating NEFA levels (Fig S2B) were similar between the *Hmgcs2*^F/F^ and *Hmgcs2*^ΔLiv^ mice. Moreover, the hepatic expression of fatty acid uptake genes such as *Cd36, Fatp2,* and *Fatp5* was not different between the genotypes (Fig. 2A). This suggests that adipose tissue lipolysis or enhanced hepatic lipid uptake mechanisms do not contribute to hepatic steatosis in *Hmgcs2*^ΔLiv^ mice. Adipose tissue-derived fatty acids activate hepatic PPARα, the nuclear transcription factor that regulates fatty acid oxidation genes^27^. Disruption of hepatic PPARα signaling induces severe steatosis after acute fasting^28^. Therefore, we assessed whether fasting-induced fatty liver in *Hmgcs2*^ΔLiv^ mice is caused by impaired PPARα signaling. As expected, fasting increased the mRNA levels of PPARα and its target genes, such as *Cpt1, Acox1, Mcad, Cpt2* and *Vcad* (Fig. 2B). However, fasted *Hmgcs2*^ΔLiv^ mice showed a similar increase in the FAO genes (Fig. 2B), suggesting that steatosis in *Hmgcs2*^ΔLiv^ mice is not caused by impaired PPARα signaling. Moreover, mRNA levels of lipogenesis-related genes such as *Srebp1, Fasn, Acc1,* and *Scd1* showed no difference between the genotypes at fed or fasted conditions (Fig. 2C). Together, our data suggest that fasting-induced hepatic steatosis in *Hmgcs2*^ΔLiv^ mice is not driven by altered lipolysis, lipid uptake, PPARα signaling, or lipogenesis.

We found that the livers of fasted *Hmgcs2*^ΔLiv^ mice expressed a significantly elevated level of fat-specific protein 27 (*Fsp27*) and perilipin (*Plin2*) (Fig. 2D). FSP27 increases lipid storage by inhibiting ATGL-mediated lipolysis^29^, while perilipin2 confers resistance of lipid droplets to lipophagy^30^. To address the involvement of lipolytic mechanisms in the liver, we assessed the expression of CGI-58 and ATGL, which showed no difference between the genotypes (Fig. 2E). We also did not find any difference in the levels of phosphorylated HSL (Fig. 2E). Lipophagy is recently recognized as a regulator of lipid droplet size and number^31^. Our analysis did not show any difference in the expression of autophagy-related proteins in fasted *Hmgcs2*^ΔLiv^ livers (Fig S2C), indicating that the lipolytic or lipophagic mechanisms in the liver are not responsible for lipid accumulation in *Hmgcs2*^ΔLiv^ mice.

Hepatic lipid content is controlled by a tight interaction between fatty acid synthesis, oxidation, and esterification^32^. Since FAO, lipogenesis, or lipolysis-related genes are unaltered, we assessed whether *Hmgcs2*^ΔLiv^ has altered lipid re-esterification. We did not find any difference in the mRNA levels of esterification-related genes such as *Dgat1, Dgat2, Gpat,* and *lipin1* between the genotypes (Fig S2D). Intriguingly, the mRNA levels of acyl-CoA synthetase long-chain family member 1 (ACSL1) were significantly elevated in the livers of fasted *Hmgcs2*^ΔLiv^ mice (Fig. 2F). However, no difference in other ACSL isoforms (Acls2, Acls3, and Acsl5) was noted (Fig. 2F). Consistent with the mRNA levels, ACSL1 protein expression was also increased in the livers of fasted *Hmgcs2*^ΔLiv^ mice (Fig. 2G). ACSL1 is a rate-limiting enzyme involved in converting fatty acids into acyl-CoA, the first step required for fatty acid oxidation or esterification^33^. To determine whether ACSL1 is responsible for steatosis in *Hmgcs2*^ΔLiv^ mice, we incubated the primary hepatocytes from *Hmgcs2*^ΔLiv^ mice and *Hmgcs2*^F/F^ mice under lipogenic condition (BSA-conjugated palmitate) in the presence or absence of Triascin C, a specific ACSL1 inhibitor^34^. The lipid accumulation was higher in the primary hepatocytes from *Hmgcs2*^ΔLiv^ mice compared to *Hmgcs2*^F/F^ (Fig. 2H and S2F). Notably, Triascin C alleviated steatosis in *Hmgcs2*^ΔLiv^ primary hepatocytes (Fig. 2H and S2E), suggesting that ASCL1 drives steatosis in *Hmgcs2*^ΔLiv^ mice.

### ACSL1 translocation to ER is regulated by ketogenesis.

The subcellular localization of ACSL1 plays a crucial role in targeting the fatty acids for catabolism or storage in the form of lipid droplets^19^. Acyl-CoA generated by the mitochondria-associated ACSL1 is channeled toward fatty acid oxidation^35^. Whereas ER-associated ACSL1 promotes fatty acid esterification to synthesize triglycerides^20^. To understand how ACSL1 induces hepatic steatosis in *Hmgcs2*^ΔLiv^ mice, we assessed the subcellular localization of ACSL1. ACSL1 is highly enriched in the isolated mitochondria of fasted livers^20^. Our data showed no difference in the mitochondrial ACSL1 protein levels between the fasted *Hmgcs2*^F/F^ and *Hmgcs2*^ΔLiv^ mice (Fig. 2I). Interestingly, we found significantly elevated ER-associated ACSL1 protein levels in the livers of fasted-*Hmgcs2*^ΔLiv^ mice (Fig. 2J). Because ER-associated ASCL1 regulates triglyceride storage via fatty acid re-esterification^20^, we performed thin-layer chromatography (TLC) to assess whether re-esterification is enhanced in *Hmgcs2*^ΔLiv^ mice. Our data showed a marked increase in the triglyceride fraction from the fasted *Hmgcs2*^ΔLiv^ mice, even after normalizing with phospholipids (Fig. 2K–M). However, no difference in phospholipids, cholesterol esters, and ceramides factions was noted (Fig. 2K and L). Together, our data indicate that ketogenesis controls the ER-ACSL1 mediated re-esterification of fatty acids under fasting conditions.

### Ketogenesis insufficiency does not affect mitochondrial function under acute fasting.

**Figure 3:**
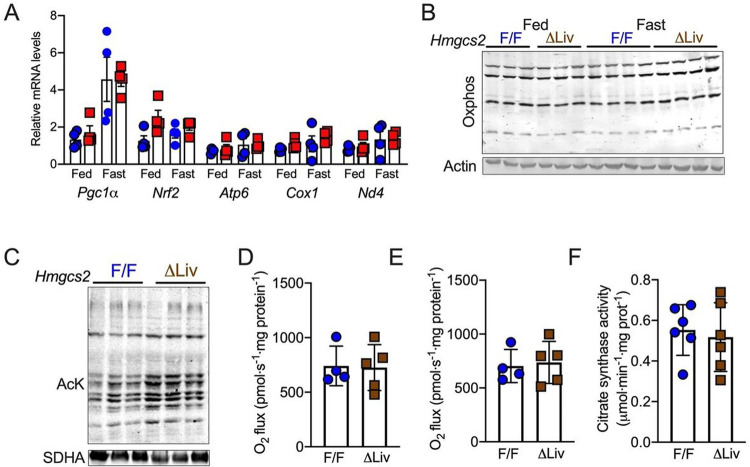
Ketogenic insufficiency does not affect mitochondrial function at acute fasting. (A) Relative mRNA levels of mitochondrial biogenesis and content-related genes in the liver of *Hmgcs2*^ΔLiv^ mice. (B) WB analysis of Oxphos proteins in the liver of *Hmgcs2*^ΔLiv^ mice. (C) WB analysis of acetyl-lysine (AcK) in the hepatic mitochondrial fractions from 16 h fasted *Hmgcs2*^ΔLiv^ mice. (D and E) Mitochondrial respiration devoted to oxidative phosphorylation or maximum electron transport chain activity in the uncoupled state. (F) Liver citrate synthase enzymatic activity. All the data is presented as mean ± SEM. Data was analyzed by One-way ANOVA (Tukey multiple-comparisons test) and Two-tailed Student *t*-test

Previous studies showed that ketone bodies are potent inducers of mitochondrial biogenesis by upregulating the expression of the transcription factor, PGC1α^36,37^. Because mitochondrial homeostasis including biogenesis and function is key in regulating lipid metabolism^38^, we assessed whether impaired ketogenesis affected mitochondrial homeostasis in *Hmgcs2*^ΔLiv^ mice. We found no difference in the mRNA expression of *Pgc1α, Nrf2,* and various other mitochondria-associated genes such as *Atp6, Cox1,* and *Nd4* in *Hmgcs2*^ΔLiv^ mice (Fig. 3A). Moreover, the expression of OXPHOS proteins such as NDUFB8, SDHB, UQCRC2, and cytochrome C oxidase was similar between the genotypes (Fig. 3B), suggesting that the temporal impairment in ketogenesis does not affect mitochondrial content. A recent study showed that TBK1 acts as a chaperone regulating the mitochondrial translocation and activity of ACSL1 under fasting conditions^20^. We found no difference between the fasted *Hmgcs2*^F/F^ and *Hmgcs2*^ΔLiv^ mice in the expression of TBK1 or its phosphorylated form (Fig S3A), indicating that the mitochondrial TBK1-ASCL1 axis may not be involved in the fasting-mediated hepatic steatosis in *Hmgcs2*^ΔLiv^ mice. Ketogenic insufficiency in neonatal pups increases the acetylation of mitochondrial proteins leading to mitochondrial dysfunction^14^. When we analyzed the isolated mitochondria, pan-protein acetylation was found to be increased in the livers of fasted *Hmgcs2*^ΔLiv^ mice (Fig. 3C). We then assessed mitochondrial respiratory capacity in the presence of palmitoylcarnitine using purified liver mitochondria from *Hmgcs2*^F/F^ and *Hmgcs2*^ΔLiv^ mice. There were no differences in mitochondrial respiration devoted to oxidative phosphorylation or maximum electron transport chain activity in the uncoupled state (Fig. 3D–E). Consistent with the data above, we also found no difference in liver citrate synthase activity (Fig. 3F), which serves as a surrogate of mitochondrial content^39^. Together, these data suggest that ketogenic insufficiency did not result in steatosis due to impaired mitochondrial fatty acid oxidation, nor did it result in compensatory increases in mitochondrial content.

### Ketogenic insufficiency exacerbates diet-induced hepatic steatosis via re-esterification.

**Figure 4:**
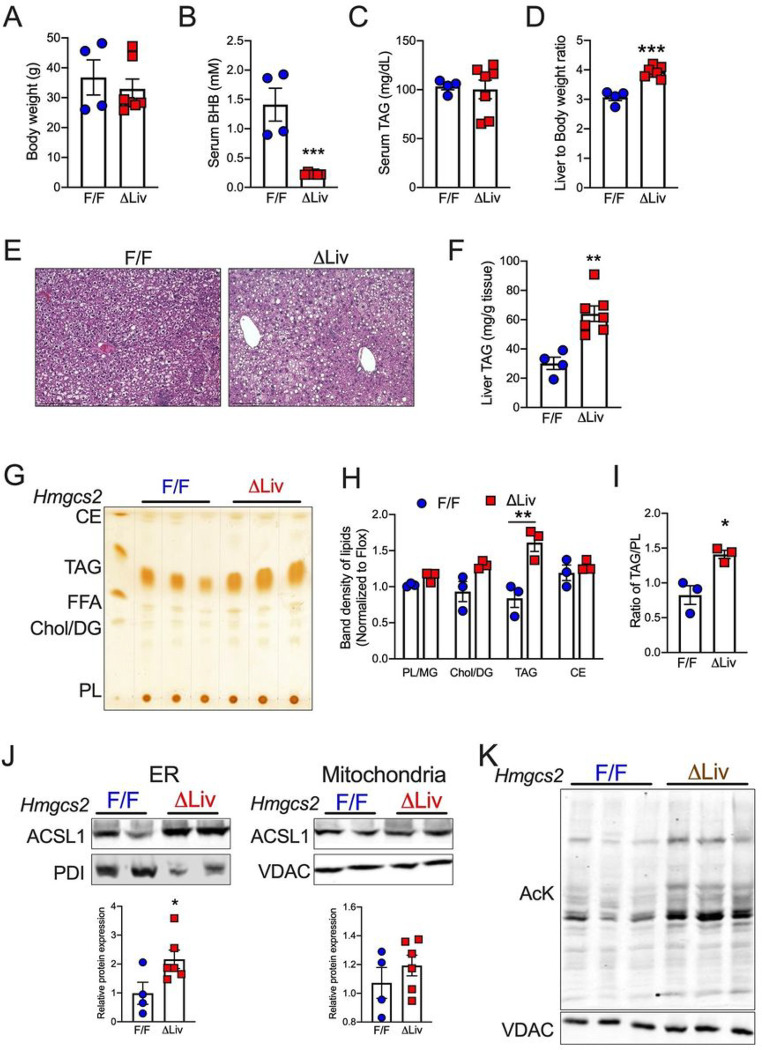
Ketogenic insufficiency exacerbates diet-induced hepatic steatosis via re-esterification. (A-D) Body weight, (B) Serum b-Hydroxybutyrate (BHB) levels, (C) Serum triglyceride (TAG) levels, and (D) Liver-to-body weight ratio in 16 h fasted *Hmgcs2*^F/F^ and *Hmgcs2*^ΔLiv^ mice fed with a 60% HFD for 4-weeks. (E) Representative H&E images and (F) TAG levels from the liver of HFD-fed fasted *Hmgcs2*^ΔLiv^ mice. (G-I) TLC image showing lipid fractions (H) Quantified band densities for lipid fractions; (I) Quantified ratio of TAG/PL in the liver of HFD-fed fasted *Hmgcs2*^ΔLiv^ mice. (J) WB analysis of ACSL1 in the hepatic mitochondrial and microsomal fractions from the liver of HFD-fed fasted *Hmgcs2*^ΔLiv^ mice. (K) WB analysis of AcK in the mitochondrial fractions. All the data is presented as mean ± SEM. p < 0.05 (*) or p < 0.01 (**) or p < 0.001(***) analyzed by Two-tailed Student *t*-test.

To understand whether hepatic ketogenesis insufficiency promotes diet-induced hepatic steatosis in response to fasting, 8-week-old HMGCS2 conditional knockout were treated with tamoxifen to disrupt HMGCS2 and then fed with 60% high-fat diet (HFD) for 4-weeks and then fasted for 16 hours before euthanasia. We found no difference in the body weight between *Hmgcs2*^F/F^ and *Hmgcs2*^ΔLiv^ mice on an HFD (Fig. 4A). As expected, ketone body levels were significantly lower in *Hmgcs2*^ΔLiv^ mice (Fig. 4B). No difference in serum triglyceride or cholesterol was noted (Fig. 4C and S4A). As in chow diet mice, the liver-to-body weight ratio was significantly higher in HFD-fed *Hmgcs2*^ΔLiv^ mice (Fig. 4D). H&E analysis showed microsteatosis in *Hmgcs2*^F/F^ mice, while *Hmgcs2*^ΔLiv^ mice showed macrosteatosis (Fig. 4E). Liver triglyceride levels were significantly increased in the HFD-fed *Hmgcs2*^ΔLiv^ mice (Fig. 4F). We assessed whether enhanced fatty acid re-esterification contributed to macrosteatosis in HFD-fed *Hmgcs2*^ΔLiv^ mice. Similar to chow-fed mice, TLC analysis showed a significant increase in triglyceride fraction in HFD-fed *Hmgcs2*^ΔLiv^ mice (Fig. 4G–I). But no difference in other lipid moieties was observed (Fig. 4G and H). We then assessed whether ER localization of ACSL1 is augmented by HFD feeding in *Hmgcs2*^ΔLiv^ mice. We found a significant increase in the ER-associated ACSL1 in HFD-fed *Hmgcs2*^ΔLiv^ mice (Fig. 4J), but no change in mitochondria-associated ACSL1 was observed (Fig. 4J). Similar to chow-fed mice, acetylated mitochondrial proteins were elevated in the mitochondria of HFD-fed *Hmgcs2*^ΔLiv^ mice (Fig. 4K). Collectively, our data show that impaired ketogenesis under nutrient-rich conditions increases ASCL1-mediated esterification of fatty acids.

### Impaired ketogenesis in human NASH is associated with increased ER-associated ACSL1 and fatty acid re-esterification.

**Figure 5:**
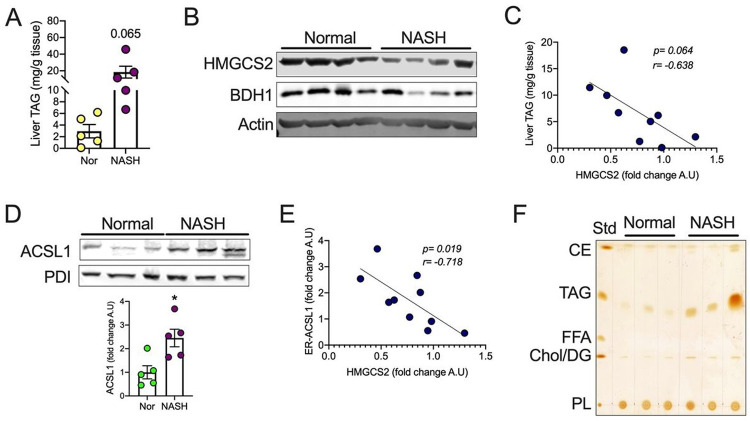
Impaired ketogenesis in human NASH is associated with increased ER-associated ACSL1 and fatty acid esterification. (A) Hepatic TAG was measured in normal and NASH subjects. (B) WB analysis for HMGCS2 and BDH1 in the livers of normal and NASH subjects. (C) Correlative analysis for liver TAG and relative HMGCS2 protein expression in normal and NASH subjects. (D) WB analysis showing hepatic ER-associated ACSL1 protein expression and (bottom) their relative quantification levels. (E) Correlative analysis for relative liver ER-associated ACSL1 and HMGCS2 protein expression in normal and NASH subjects (F) TLC image showing lipid fractions from liver tissues. All the data is presented as mean ± SEM. Correlative analysis was performed by using a non-parametric Spearman’s test. p < 0.05 (*) analyzed by the Two-tailed Student *t*-test.

NASH is the most common liver disease, where excess and sustained lipid accumulation (steatosis) triggers a myriad of pathological changes culminating in liver inflammation, fibrosis, and cancer^3^. Hepatic steatosis is majorly driven by a mismatch in the delivery (excessive lipolysis and dietary intake) and handling of lipids (fatty acid synthesis, oxidation, storage, and secretion) by the hepatocytes^5^. When the flux of fatty acids is acute, a compensatory increase in FAO and ketogenesis protects the liver from lipotoxicity^40^. However, chronic steatotic conditions decrease FAO and ketogenesis resulting in the accumulation of lipid droplets. Indeed, studies show that ketone body levels decline progressively in NASH patients^13,41,42^. Based on our results, we investigated whether hepatic ketogenesis is associated with elevated fatty acid esterification in NASH patients. To this end, we confirmed higher triglyceride levels in deidentified liver tissues from NASH patients undergoing surgical resection compared to control human (normal) subjects (Fig. 5A). Our analysis showed no difference in serum triglyceride levels but BHB levels trended lower in NASH patients (Fig S5A and B). Further, the liver from NASH patients showed a significant decrease in the protein levels of HMGCS2 and 3-hydroxybutyrate dehydrogenase (BDH1), the latter catalyzes the interconversion of β-hydroxybutyrate and acetoacetate (Fig. 5B and S5C). A correlation analysis shows that triglyceride levels increase as the hepatic expression of HMGCS2 decreases (Fig. 5C). Since ketogenesis is impaired in NASH, we asked whether ASCL1 translocation is modulated in the NASH livers. We found significantly elevated levels of ER-associated ACSL1 in NASH subjects (Fig. 5D). Moreover, we observed a negative correlation between the ER-ACLS1 and HMGCS2 protein expression (Fig. 5E); however, no difference in the mitochondria-associated ACLS1 was noticed (Fig S5D). TLC analysis showed increased levels of esterified triglyceride compared to other lipid fractions (Fig. 5F and S5E), suggesting that impaired ketogenesis is associated with increased ER-ASCL1 mediated re-esterification of lipids in NASH patients. Similar to mouse models, impaired ketogenesis is also associated with elevated levels of acetylated mitochondrial proteins in human NASH livers (Fig S5F). Collectively, our data show that impaired ketogenesis induces lipid accumulation in NASH patients in part by promoting the ASCL1-mediated re-esterification of fatty acids.

### Hepatic acetyl-CoA-L-carnitine homeostasis regulates lipid partitioning via ACSL1.

**Figure 6:**
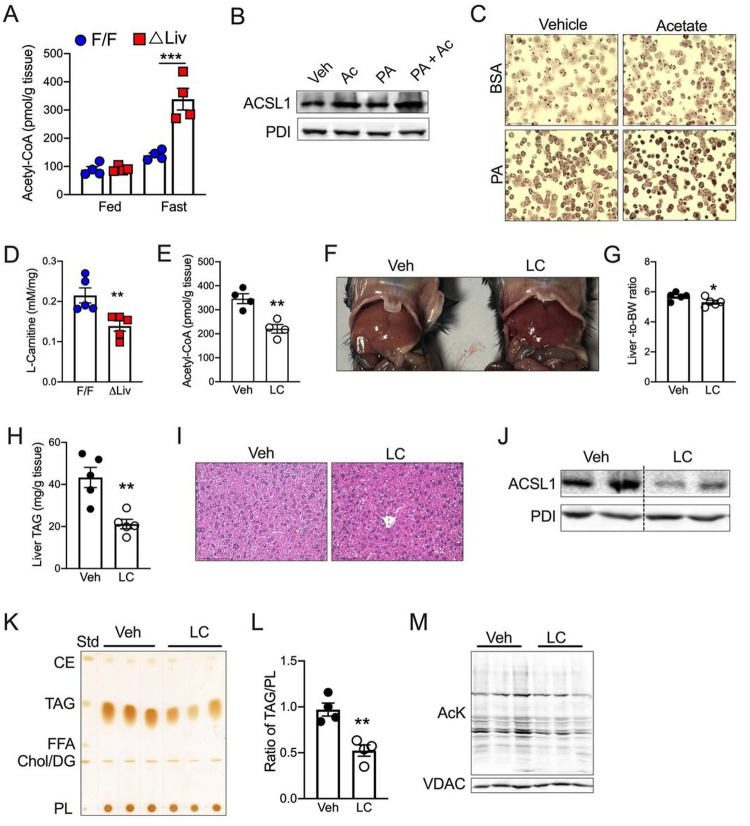
Hepatic acetyl-CoA-L-carnitine homeostasis regulates lipid partitioning via ACSL1 (A) Liver acetyl-CoA levels in fed and fasted *Hmgcs2*^ΔLiv^ mice. (B) WB analysis of ER-ACSL1 in primary hepatocytes incubated with 200mM BSA-conjugated PA in the presence or absence of 20 mM sodium acetate for 16 h. (C) Oil-red-O images showing lipid-laden primary hepatocytes. (D) Liver L-carnitine (LC) levels were measured in fasted *Hmgcs2*^ΔLiv^ mice. (E) Liver acetyl-CoA levels from fasted *Hmgcs2*^ΔLiv^ mice treated with or without LC (10mg/ml). (F) Gross liver image, (G) liver-to-body weight, (H) liver TAG, and (I) H&E analysis of the livers from fasted *Hmgcs2*^ΔLiv^ mice treated with or without LC. (J) WB analysis showing ER-ACSL1 protein levels from the livers of fasted *Hmgcs2*^ΔLiv^ mice treated with or without LC. (K) TLC and (L) TAG quantification in the livers of fasted *Hmgcs2*^ΔLiv^ mice treated with or without LC. (M) WB analysis for AcK in the mitochondrial fraction of fasted *Hmgcs2*^ΔLiv^ mice treated with or without LC. All the data is presented as mean ± SEM. p < 0.05 (*); or p < 0.01 (**) or p < 0.001(***) analyzed by Two-tailed Student t test.

We then sought to understand how ketogenesis regulates the ER translocation of ACSL1. Ketogenesis is the major route of eliminating acetyl-CoA generated by FAO, though some amount of acetyl-CoA would be metabolized via the TCA cycle, or exported out of mitochondria as citrate^12,43^. We found no difference in the acetyl-CoA levels under fed conditions but fasting increased the acetyl-CoA levels by 2-fold in the livers of *Hmgcs2*^ΔLiv^ mice (Fig. 6A). Based on the coincidental increase in acetyl-CoA and ER-associated ACSL1 in fasted *Hmgcs2*^ΔLiv^ mice, we posited that acetyl-CoA might regulate the subcellular localization of ACSL1. To test our hypothesis, we incubated mouse primary hepatocytes under lipogenic conditions with or without acetate, which increases intracellular acetyl-CoA levels. Western blot analysis showed that acetate increases ER-associated ACSL1 both in the presence and absence of palmitic acid (Fig. 6B and S6A). Further, acetate increased lipid accumulation in primary hepatocytes (Fig. 6C and S6B). This data suggests that elevated acetyl-CoA induces ER translocation of ACSL1 and exacerbates steatosis under lipogenic conditions.

We then interrogated ways to decrease acetyl-CoA levels as a resort to alleviate hepatic steatosis under conditions of impaired ketogenesis. L-carnitine (LC) acts as a buffer for mitochondrial acetyl-CoA by forming acetyl-carnitine, i.e., excess mitochondrial acetyl-CoA exists in the form of acetyl-carnitine^44^. LC is also required for the mitochondrial import of acyl-CoA for FAO. Thus, excess mitochondrial acetyl-CoA reduces mitochondrial FAO by decreasing free LC levels^21,45^. Our analysis showed significantly reduced free LC levels in the livers of fasted *Hmgcs2*^ΔLiv^ mice (Fig. 6D). We hypothesized that increasing LC would restore lipid homeostasis in *Hmgcs2*^ΔLiv^ mice by decreasing hepatic acetyl-CoA levels. To this end, we supplemented the drinking water with LC @ 10mg/ml and fasted the *Hmgcs2*^ΔLiv^ mice for 16 hours. No change in body weight was observed by LC supplementation (Fig S6C). LC supplementation reduced acetyl-CoA levels in the fasted *Hmgcs2*^ΔLiv^ mice (Fig. 6E). Notably, the livers of LC-treated *Hmgcs2*^ΔLiv^ mice were reddish compared to untreated littermates (Fig. 6F). The liver-to-body weight ratio was significantly lower and the liver triglyceride levels were significantly reduced in LC-treated fasted *Hmgcs2*^ΔLiv^ mice (Fig. 6G and H). H&E analysis confirmed the reduction in hepatic steatosis in LC-treated fasted *Hmgcs2*^ΔLiv^ mice (Fig. 6I). We further demonstrate that LC supplementation decreased the ER localization of ACSL1 (Fig. 6J), resulting in reduced esterification of triglyceride in the fasted *Hmgcs2*^ΔLiv^ mice (Fig. 6K and L, and S6D). We further noticed that LC decreased the levels of acetylated mitochondrial proteins in fasted *Hmgcs2*^ΔLiv^ mice (Fig. 6M). Together, our data indicate that LC recuperates acetyl-CoA homeostasis and alleviates ER-ACSL1-mediated hepatic steatosis in mice with impaired ketogenesis.

### L-carnitine alleviates steatosis in human NASH partly by reducing acetyl-CoA and ER-associated ASCL1.

**Figure 7:**
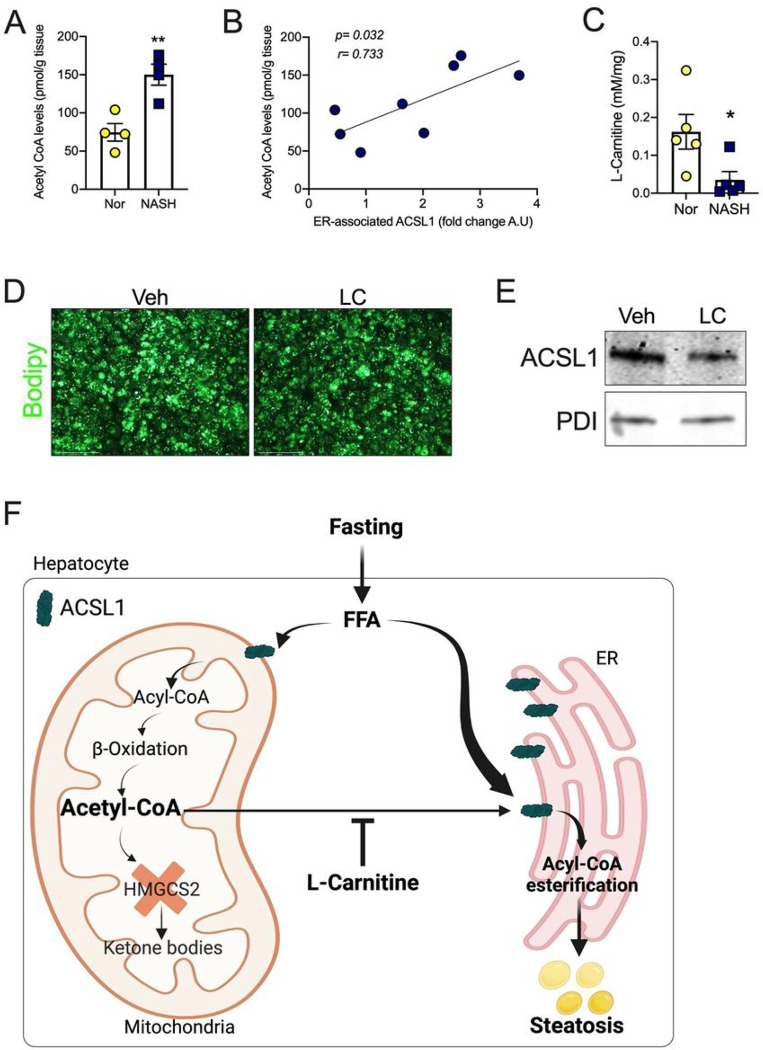
L-carnitine alleviates steatosis in human NASH partly by reducing ASCL1-mediated esterification of fatty acids. (A) Acetyl-CoA levels were measured in the livers of normal and NASH subjects. (B) Correlation analysis for liver acetyl-CoA levels and relative protein expression of hepatic ER-localized ACSL1. (C) Liver L-carnitine levels. (D) Images of BODIPY stained primary human hepatocytes from NASH subjects pre-treated with 1mM L-carnitine for 6 h and loaded with 200mm BSA-PA for 16 h (20X magnification). (E) WB analysis of ACSL1 in the microsomal fraction of primary human hepatocytes from NASH subjects pre-treated with 1mM L-carnitine for 6 h and loaded with 200mm BSA-PA for 16 h. (F) Schematic diagram. All the data is presented as mean ± SEM. Correlative analysis was performed by using a non-parametric Spearman’s test. p < 0.05 (*) or p < 0.01 (**) analyzed by the Two-tailed Student *t*-test.

We then evaluated the relationship between acetyl-CoA and ACSL1-mediated esterification in the livers of NASH patients. As shown in previous studies^42,46^, liver acetyl-CoA levels were elevated in the NASH subjects (Fig. 7A). Correlative analysis showed that ER-associated ACSL1 was in proportion with acetyl-CoA levels in human NASH livers (Fig. 7B). Similar to the mouse models, free L-carnitine (LC) levels were significantly reduced in NASH livers and were positively correlated with hepatic HMGCS2 expression (Fig. 7C and S7A). Previous studies have demonstrated that LC improves steatosis in NASH patients by increasing mitochondrial function and FAO^47,48^. We tested whether LC affects ACSL1-mediated fatty acid esterification using primary hepatocytes from NASH patients. BODIPY staining showed reduced lipid accumulation with LC treatment in primary human NASH hepatocytes cultured under lipogenic conditions (Fig. 7D). We demonstrate that LC decreases the ER-associated ACSL1 (Fig. 7E) and fatty acid esterification in human NASH primary hepatocytes (Fig S7B). In contrast to mouse livers, LC increased ASCL1 levels in the mitochondria, and the levels of acetylated mitochondrial proteins were decreased (Fig S7C and D). Collectively, we demonstrate that LC alleviates steatosis by reducing acetyl-CoA levels and ER translocation of ACSL1 in human NASH.

## Discussion

Perturbation in hepatic fatty acid metabolism is the key risk factor driving several metabolic diseases, including obesity, diabetes, and NAFLD. An increase in free fatty acid influx from peripheral tissue, augmented lipid biosynthesis, and impaired lipid disposal in the liver lead to triglyceride accumulation triggering hepatic steatosis. Lipid disposal pathways, in particular β-oxidation play an essential role in eliminating excessive hepatic lipids under physiological and pathological conditions^40,49,50^. Indeed, under fasting conditions, the liver disposes up to 250g of lipid per day through β-oxidation and ketogenesis^51^. However, it remains unclear whether hepatic ketogenesis is just a regulator of peripheral energy homeostasis or has a role in regulating hepatic lipid metabolism. We show that the mouse with temporal disruption of hepatic ketogenesis develops severe steatosis at fasting. While there is no change in fatty acid uptake, *de novo* lipogenesis, and β-oxidation pathways, ketogenesis is found to regulate fatty acid partitioning via ER-associated ACSL1. We show that the accumulation of acetyl-CoA in response to ketogenic insufficiency induces ER translocation of ACSL1 and fatty acid esterification. Thus, incoming fatty acids are mistargeted for storage but not FAO, as elevated acetyl-CoA (from ketogenic insufficiency) is misinterpreted as excessive FAO. We demonstrate that L-carnitine supplementation in ketogenic insufficiency mice counteracts the acetyl-CoA-mediated increase in ER-associated ACSL1 and triglyceride esterification. We also report elevated acetyl-CoA, ER-associated ACSL1, and enhanced triglyceride esterification in NASH patients eliciting impaired ketogenesis. Thus, we define a novel role of ketogenesis in regulating hepatic lipid metabolism.

Recent studies demonstrated a causal role of ketogenesis in fatty liver pathogenesis using neonates and adult mice with ketogenic insufficiency (via disruption of hepatic HMGCS2)^14,15^. These mice develop fatty liver when fed lipid-rich mothers’ milk and a high-fat diet^15^. We show that acute fasting is sufficient to induce steatosis in ketogenesis insufficient mice. Despite the availability of food ad-lib, mice tend to eat at night, i.e. they undergo physiological fasting at day time. No difference in liver triglyceride levels in ad-lib-fed ketogenesis insufficient mice indicates that a small increase in fatty acids from physiological fasting is well-tolerated. Refeeding induces the secretion of hepatic triglycerides as VLDL, which are taken up by peripheral tissues, including adipose tissue, heart, kidney, and vascular tissue^50^. Previous studies showed that ketogenesis regulates the composition of hepatic lipids^16,52^; therefore, investigating the impact of ketogenic insufficiency on peripheral lipid homeostasis may shed light on the mechanistic underpinnings of metabolic diseases, including cardiovascular disease.

Fasting-mediated adipose tissue lipolysis and a drop-in insulin levels coordinate to induce hepatic ketogenesis. Thus, inhibition of adipose tissue ATGL decreases the circulating ketone body levels^27^. Under these circumstances, ketone body levels are tightly linked to fatty acid oxidation, evident from PPARα knockout mice eliciting diminished fasting-associated ketogenesis despite no difference in circulating fatty acids^28^. Lipid accumulation in ketogenesis insufficiency mice was not due to impairment in hepatic PPARα signaling or lipogenic pathways. Our data show increased fatty acid storage evident from microsteatosis and increased expression of lipid droplet-associated proteins such as Plin2 and FSP27. This led us to speculate that ketogenetic insufficiency promotes lipid accumulation via the esterification of fatty acids.

Free fatty acids entering the hepatocytes are immediately esterified into acyl CoA by ACSL1. ACLS1 in the outer mitochondria membrane directs acyl-CoA for β-oxidation^19,20^, whereas ER-localized ACSL1 generates lipid substrates for triglyceride synthesis via re-esterification^49^. Nonetheless, the mechanisms regulating the ER translocation of ACSL1 are unclear. We demonstrate that ketogenesis is tightly linked to ER localization of ACSL1 in fasted mice. The significance of ER-ACSL1 in triglyceride synthesis via re-esterification is also established in human NASH livers, where the translocation of ACSL1 to ER-localized is dramatically increased and is strongly associated with lower expression of HMGCS2. Thus, our data suggest that impairment in hepatic ketogenesis increases ER localization of ACSL1 in both human and mouse NASH models.

Acetyl-CoA acts as a metabolic node regulating glucose, lipid, and amino acid metabolism^53^. Acetyl-CoA levels increase during fasting and high-fat feeding due to elevated FAO^54^. The conduit of acetyl-CoA as ketone bodies is necessary to maintain cellular acetyl-CoA levels. When FAO exceeds ketogenesis, the increase in acetyl-CoA pauses mitochondrial FAO by acetylating the mitochondrial proteins^55,56^. Until acetyl-CoA homeostasis recuperates, the fatty acids are channeled toward ER for storage as triglycerides. Despite an increase in the acetylation of mitochondrial proteins, mitochondrial function was similar between the genotypes suggesting that mitochondrial dysfunction is not the primary cause of lipid accumulation in acute fasted ketogenic insufficient mice. Excess acetyl-CoA shuttles to the cytosol as citrate, where cytosolic ATP citrate lyase (ACLY) breaks down citrate into acetyl-CoA and oxaloacetate^53^. Since ACLY expression is upregulated in the fasted ketogenic insufficient mice, we interrogated whether acetyl-CoA spillover into the cytosol acts as a signal for the ER translocation of ACSL and lipid esterification. Acetyl-CoA levels could be increased by treating with the short-chain fatty acid acetate, which is metabolized by the enzyme acetyl-coenzyme A synthetase to generate cytosolic acetyl-coA^57^. Our data that acetate increases ER-translocation of ACSL1 and lipid-laden hepatocytes support the hypothesis that cytosolic acetyl-CoA promotes fatty liver by inducing the esterification of lipids via ACSL1.

Mitochondrial membranes are impermeable to fatty acyl-CoA; therefore, fatty acyl-CoA is esterified with L-carnitine to form acyl-carnitine by the mitochondrial enzyme CPT1. In the mitochondrial matrix, CPTII releases the free carnitine from acyl-carnitine forming acyl-CoA. The free L-carnitine is then exported back to the cytosol to participate in mitochondrial fatty acid transport. L-carnitine also buffers acetyl-CoA by forming acetyl-carnitine, which is excreted in the urine^58,59^. Studies show that carnitine levels decrease in the latter state of liver disease^22,60^, indicating that carnitine deficiency may not trigger hepatic steatosis but could exacerbate disease progression. However, patients with primary carnitine deficiency due to a lack of organic cation/carnitine transporter develop hepatomegaly and hepatic steatosis, which could be alleviated by L-carnitine supplementation^61–63^. Moreover, studies demonstrate that L-carnitine is rate-limiting for ketogenesis from endogenous fatty acids. For instance, ketosis with carnitine supplementation improves glucose homeostasis, insulin sensitivity, and lipid profile^64,65^. Thus, carnitine levels are a key determinant of lipid metabolism, ketogenesis, and hepatic steatosis. Here, we demonstrate that carnitine supplementation attenuates hepatic steatosis under ketogenic insufficiency by alleviating ER translocation of ACSL1. Carnitine also improves mitochondrial function by decreasing the levels of acetylated mitochondrial proteins^44^. No export mechanism exists to excrete cellular acetyl-CoA. We posit that carnitine, via forming acetyl-carnitine, acts as a conduit to remove excess acetyl-coA. Several studies have demonstrated the beneficial anti-inflammatory role of acetyl-carnitine in metabolic diseases^66,67^. Further investigation is required to determine whether carnitine sequestration as acetyl-carnitine mediates the anti-inflammatory response in NASH patients exhibiting impaired ketogenesis.

While it is true that ketogenesis is a selfless function of the liver in providing nutrients to other organs^12^, our data show that the conduit of acetyl-CoA via ketogenesis is crucial to regulate mitochondrial acetylome, ER homeostasis, and lipid partitioning in the liver. Dysregulated acetyl-CoA induces hepatic steatosis via ACSL1-mediated TG synthesis in the ER^19,20,68^. This mechanism particularly plays a crucial role in conditions of increased hepatic fatty acid inflow, such as fasting, overnutrition, and fatty liver disease. Although pharmacological inhibition of ACSL1 ameliorates steatosis^18,20^, the risk of liver injury is enhanced by the accumulating free fatty acids^69^. We provide empirical evidence that L-carnitine inhibits ER translocation of ACSL1 and hepatic steatosis by buffering acetyl-CoA. However, L-carnitine should be recommended with utmost care as carnitine metabolism in the intestine generates TMAO, a metabolite with atherogenic and carcinogenic properties^70,71^. Our study provides a rationale for the therapeutic use of L-carnitine in a subset of NAFLD patients exhibiting lower L-carnitine and ketone bodies. As carnitine is excreted as acetyl-carnitine^72^, therapeutic interventions with L-carnitine could be monitored non-invasively in NASH patients by measuring the urinary acetyl-carnitine levels.
